# *Mycobacterium ulcerans* Ecological Dynamics and Its Association with Freshwater Ecosystems and Aquatic Communities: Results from a 12-Month Environmental Survey in Cameroon

**DOI:** 10.1371/journal.pntd.0002879

**Published:** 2014-05-15

**Authors:** Andrés Garchitorena, Benjamin Roche, Roger Kamgang, Joachim Ossomba, Jérémie Babonneau, Jordi Landier, Arnaud Fontanet, Antoine Flahault, Sara Eyangoh, Jean-François Guégan, Laurent Marsollier

**Affiliations:** 1 UMR MIVEGEC 5290 CNRS - IRD - Université de Montpellier I - Université de Montpellier II, Montpellier, France; 2 Ecole des Hautes Etudes en Santé Publique, Rennes, France; 3 Service de Mycobactériologie, Centre Pasteur du Cameroun, Réseau International des Instituts Pasteur, Yaoundé, Cameroun; 4 UMMISCO, UMI IRD-UPMC 209, Bondy, France; 5 ATOMycA, Inserm Avenir Team, CRCNA, Inserm U892, 6299 CNRS and LUNAM, CHU and Université d'Angers, Angers, France; 6 Institut Pasteur, Unité d'Epidemiologie de Maladies Emergentes, Paris, France; 7 Conservatoire National des Arts et Métiers, Paris, France; 8 Centre Virchow-Villermé, Descartes School of Medicine, Université Sorbonne Paris-Cité, Paris, France; 9 Global Health Institute, School of Medicine, University of Geneva, Geneva, Switzerland; Institut Pasteur, France

## Abstract

**Background:**

*Mycobacterium ulcerans* (MU) is the agent responsible for Buruli Ulcer (BU), an emerging skin disease with dramatic socioeconomic and health outcomes, especially in rural settings. BU emergence and distribution is linked to aquatic ecosystems in tropical and subtropical countries, especially to swampy and flooded areas. Aquatic animal organisms are likely to play a role either as host reservoirs or vectors of the bacilli. However, information on *MU* ecological dynamics, both in space and time, is dramatically lacking. As a result, the ecology of the disease agent, and consequently its mode of transmission, remains largely unknown, which jeopardizes public health attempts for its control. The objective of this study was to gain insight on *MU* environmental distribution and colonization of aquatic organisms through time.

**Methodology/Principal Findings:**

Longitudinal sampling of 32 communities of aquatic macro-invertebrates and vertebrates was conducted from different environments in two BU endemic regions in Cameroon during 12 months. As a result, 238,496 individuals were classified and *MU* presence was assessed by qPCR in 3,084 sample-pools containing these aquatic organisms. Our study showed a broad distribution of *MU* in all ecosystems and taxonomic groups, with important regional differences in its occurrence. Colonization dynamics fluctuated along the year, with the highest peaks in August and October. The large variations observed in the colonization dynamics of different taxonomic groups and aquatic ecosystems suggest that the trends shown here are the result of complex ecological processes that need further investigation.

**Conclusion/Perspectives:**

This is the largest field study on *MU* ecology to date, providing the first detailed description of its spatio-temporal dynamics in different aquatic ecosystems within BU endemic regions. We argue that coupling this data with fine-scale epidemiological data through statistical and mathematical models will provide a major step forward in the understanding of *MU* ecology and mode of transmission.

## Author Summary

Buruli ulcer, caused by the pathogen *Mycobacterium ulcerans (MU)*, is a severe skin disease occurring in tropical and subtropical countries. Strongly associated to freshwater ecosystems and especially swampy and flooded areas, transmission of this bacterium within ecosystems and across multiple aquatic organisms is still an enigma. Here, we studied in depth the temporal and spatial variations of *MU* presence in freshwater ecosystems and aquatic organisms in two areas of Cameroon where Buruli ulcer is endemic. We found *MU* widely present across ecosystems and taxonomic groups along the year and we described a general trend for *MU* persistence in the environment. Moreover, the colonization dynamics of aquatic ecosystems suggest that each kind of ecosystem may have distinct favourable times of the year for *MU* presence. In addition to setting the scene for a preventive approach for humans based on ecosystem characteristics, this study suggests that *MU* transmission is the result of complex ecological processes between biotic and environmental factors. Such results call for an integrative approach in order to disentangle the respective contributions of aquatic organisms and environmental conditions on *MU* presence and persistence in the environment.

## Introduction

*Mycobacterium ulcerans (MU)* is the agent responsible of Buruli ulcer (BU), an emerging human skin disease affecting human populations in tropical and subtropical regions [1]. While effective treatment is available through a combination of rifampicin-streptomycin for small lesions, with additional surgery required in some cases, early access to treatment is often an issue, especially in poor rural areas where most of the disease burden accumulates [2]–[4]. Absence or delay of treatment may cause irreversible deformity, long-term disabilities, extensive skin lesions, and even severe secondary infections [5]. Public health efforts for disease control require early detection of cases, but MU ecology and the conditions triggering human infection are poorly understood, which undermines our capacity to detect areas at risk.

Buruli ulcer has been present in Cameroon since the first reported cases in 1969 from the Centre Province, in the districts of Akonolinga and Ayos [6]. The highest BU prevalence in this region dominated by tropical rainforest is distributed along the Nyong River basin, where swampy and flooded areas prevail [7]. A second endemic site appeared in 2004 in Bankim (Adamaoua Province), a region at the border with Nigeria in a transition zone between forest and savannah. Within this region, the construction of a dam in 1989 resulted in a large area of flooded land in the district, and BU cases are mostly concentrated between this dam and the Mbam River [8].

Distribution of human cases around the world seems to be closely related to freshwater ecosystems, especially to areas of slow flowing or stagnant waters [9]–[14]. Furthermore, emergence of cases in many parts of the world has been associated to the creation of swamps and flooded areas either naturally after heavy rains [13] or under the pressure of human action, *i.e.* construction of dams or irrigation [8], [15]. Micro-aerobic conditions may promote *MU* growth [16] and genomic analyses suggest that *MU* has adapted to a restricted environmental niche, possibly an arthropod [17]–[19]. Favorable conditions in these types of environment are likely to drive *MU* growth and persistence and may ultimately affect the transmission to human populations. A direct transmission could take place from the environment where *MU* is present through existing wounds or passive inoculation [20]–[23]. However, a direct link between the type of ecosystem and *MU* abundance in the environment has never been shown.

The role of aquatic communities of macro-invertebrates and vertebrates as a fundamental part of the aquatic ecosystem on *MU* ecology and transmission is also unclear. Following detection of *MU* in the environment from abiotic, *i.e.* water, soil [24], [25] and biotic samples, *i.e.* plants, fishes [26], tadpoles [27], insect larvae [28], snails [29], and water bugs [30], it has been suggested that bacteria present in the aquatic environment (water, plant biofilm, mud, and detritus) could be concentrated by filtering and grazing invertebrates and then be transmitted through predation up to higher levels of the aquatic trophic web [31]. In addition, some specific taxonomic groups could act as keystone species in the transmission of *MU* within the aquatic ecosystem [32]. Finally, water bugs of the families Belostomatidae and Naucoridae (Order Hemiptera), which are voracious predators of aquatic organisms may get colonized through this trophic web and transmit the bacteria to humans through biting [30], [33]–[35].

In order to better understand such a complex disease system, it is essential to address its changes over time and space. Freshwater ecosystems are highly dynamic with seasonal variations in abiotic and biotic parameters impacting on aquatic community assemblages and structures [36], [37]. However, comprehensive field studies performed in Africa to date have addressed temporal dynamics but in only one taxonomic order, Hemiptera water bugs [38], or have focused on aquatic communities but neglecting their temporal dimension [39], [40]. As a result, detailed information on temporal dynamics of *MU* persistence and spread in the whole aquatic community is dramatically lacking.

Here, we address this issue by performing a large-scale sampling of multiple aquatic communities over space and time in two BU endemic areas of Cameroon, Akonolinga and Bankim. This study aims to improve knowledge on *MU* environmental distribution and colonization of aquatic organisms throughout the year, with two specific objectives:

1. To compare *MU* spatio-temporal distribution in various aquatic ecosystems including swamps, flooded areas, rivers and streams, from two regions with distinct environmental characteristics.
2. To characterize *MU* colonization of aquatic communities of macro-invertebrates and vertebrates and its temporal dynamics along the year.

## Materials and Methods

### Study area and sample sites

Between June 2012 and May 2013, periodic sampling of aquatic communities was performed in Akonolinga and Bankim, two regions in Cameroon where BU is endemic [7], [8]. In order to track colonization dynamics, monthly samples were collected in Akonolinga. In addition, sampling was performed every three months in Bankim, allowing a description of a wider range of environmental characteristics (savannah and tropical rainforest). Within each region, selection of survey sites was done in a two-step procedure. Initially, we classified the villages in each region based on (i) BU human prevalence and (ii) surrounding environmental conditions, according to national health data and land cover data respectively. We pre-selected a number of villages that represented a gradient in both of these parameters within each region. In order to evaluate the relevance of these sites for the study, this pre-selection was followed by on-site visits of all water bodies surrounding the villages and discussions with the local population and health authorities (accessibility, land-use change, human use, persistence throughout the year, etc.).

In all, 32 water sites were selected (16 in each region), including a large variety of streams, rivers, swamps and flooded areas. Streams were defined as bodies of water with a current and were clearly confined within a bed of up to 30 m wide. They included both rainforest streams in Akonolinga and rainforest and savannah streams in Bankim. Rivers were larger than streams, and their margin was highly variable depending on the season, being up to several hundred meters wide in periods of intensive rainfall. They included the Nyong and Mfoumou rivers in Akonolinga, but not the Mbam river in Bankim due to very strong currents that prevented appropriate sampling. We considered as swamps all permanent wetlands with stagnant or very slow flowing waters, many of which were created as the result of roads blocking the natural course of a stream. Finally, flooded areas were temporary bodies of stagnant water formed either naturally after heavy rains in flat areas of forest or savannah, or artificially as in the case of the Mapé Dam in Bankim.

### Aquatic sampling

Sampling in each region was performed between 8am and 4pm during 5 consecutive days. In order to ensure comparability of the results, identical methods were carried out by the same persons for all sites throughout the study. In each water body, 4 locations were chosen in areas of slow water flow and among the dominant aquatic vegetation. The sample was limited to those places accessible by a person with waders (depth max. 1.50 m). At each location, 5 sweeps were done with a metallic dip net (32×32 cm, 1 mm mesh size) within a surface of 1 m^2^ and at different depth levels (down to a depth of 1 m). All the material collected was placed into a bucket with water and passed through a 3-layer filter (32×32 cm grid; 20, 5 and 1 mm mesh sizes, respectively) with abundant water. The material in the first two layers was placed in white rectangular basins, and visible aquatic organisms were identified on site, classified and stored separately into tubes with 70% ethanol. The material contained in the last layer, a mixture of plant debris and small invertebrates, was put into 150 ml flasks with 95% ethanol and brought to the laboratory, where identification of all other individuals in the community (larger than 1 mm) was done with the use of a binocular microscope.

### Entomological classification and PCR pool design

Aquatic macro-invertebrates were classified down to the family level whenever possible, using taxonomic keys provided in the Guide to the Freshwater Invertebrates of Southern Africa series [41]–[47] and other relevant literature [48]–[51]. In order to avoid cross-contamination between samples, all the equipment used in the classification (forceps, basins, gloves, Petri dishes, etc.) was discarded or decontaminated with NaOH 1 M at the end of each sample classification.

Individuals from the same sample were pooled for PCR analysis by groups of aquatic organisms belonging to the same taxonomic group. Two pooling strategies were used to fulfill the purposes of our study. First, for all sites, we tested a total of 6 sample-pools for each month and each site in order to better describe spatio-temporal dynamics of *MU* presence. For this, we chose the 5 most abundant taxonomic groups in all sites (to allow for comparability of results) plus a sixth group that was different in each site (to gain representation of all groups), and we pooled all individuals of the same group. Second, we chose 10 sites, 5 sites in each region, for which we applied a more in-depth molecular analysis every 3 months in order to have a better characterization of *MU* presence in taxonomic groups. Within these sites, all individuals of each taxonomic group were distributed in 4 sample-pools, and all taxonomic groups were tested. The same 10 sites were used along the year and this subgroup presented a similar geographical and environmental variability as the larger group of 32 sites. A maximum of 2 g of pool weight was established in order to avoid excessive inhibition during the qPCR analysis. For each sample-pool, composition, number of individuals and weight of the pool were recorded.

### DNA extraction and purification from pools of aquatic organisms

Pooled individuals were all ground together and homogenized in 50 mM NaOH solution using Tissue Lyser II (QIAGEN). Tissue homogenates were heated at 95°C for 20 min. DNA from homogenized insect tissues was purified using QIAquick 96 PCR Purification Kit (QIAGEN), according to manufacturer's recommendations. 10% negative controls were included for extraction and purification.

### Detection of *MU* DNA by quantitative PCR

Oligonucleotide primer and TaqMan probe sequences were selected from the GenBank IS*2404* sequence [52] and the ketoreductase B (KR) domain of the mycolactone polyketide synthase (mls) gene (Table 1) from the plasmid pMUM001 [52], [53]. QPCR mixtures contained 5 µl of template DNA, 0.3 µM concentration of each primer, 0.25 µM concentration of the probe, and Brilliant II QPCR master Mix Low Rox (Agilent Technologies) in a total volume of 25 µl. Amplification and detection were performed with Thermocycler (Chromo 4, Bio-Rad) using the following program: 1 cycle of 50°C for 2 min, 1 cycle of 95°C for 15 min, 40 cycles of 95°C for 15 s and 60°C for 1 min. DNA extracts were tested at least in duplicates and the 10% negative controls were included in each assay. Quantitative readout assays were set up, based on external standard curve with MU (strain 1G897) DNA serially diluted over 5 logs (from 10^6^ to 10^2^ U/ml). Samples were considered positive only if both the gene sequence encoding the ketoreductase B domain (KR) of the mycolactone polyketide synthase and IS*2404* sequence were detected, with threshold cycle (Ct) values strictly <35 cycles.

**Table 1:** Primers and probes used to detect *M. ulcerans* DNA sequences by Taq Man real-time PCR.

Primer or Probe Name	Sequence (5′ to 3′)
KR-B forward primer	TCACGGCCTGCGATATCA
KR-B reverse primer	TTGTGTGGGCACTGAATTGAC
KR-B probe	FAM-ACCCCGAAGCACTGGCCGC-TAMRA
IS2404 forward primer	ATTGGTGCCGATCGAGTTG
IS2404 reverse primer	TCGCTTTGGCGCGTAAA
IS2404 probe	FAM-CACCACGCAGCATTCTTGCCGT-TAMRA

### Data analysis

All statistical analyses were conducted using *R* statistical software, version 2.14.0 [54]. Maps were created using ArcGIS 10.0 and information displayed in them was obtained from the USGS Shuttle Radar Topography Mission (elevation data) [55], IFORA project (hydrographic network) and Institut National de Cartographie du Cameroun (roads). Data on rainfall was obtained from the NASA Tropical Rainfall Measuring Mission [56]. Pearson Chi Square tests were used to compare proportions of positive sample-pools coming from different types of ecosystems and p-values were computed by Monte-Carlo simulation. One-sample proportions tests with continuity correction were used to calculate the confidence intervals of the proportions. Associations of *MU* colonization dynamics of taxonomic groups or *MU* colonization of different ecosystems with rainfall patterns were investigated by calculating the cross-correlation of the time series two by two.

## Results

### Global distribution of *M. ulcerans*

#### Distribution of *M. ulcerans* in aquatic ecosystems

**Figure 1:**
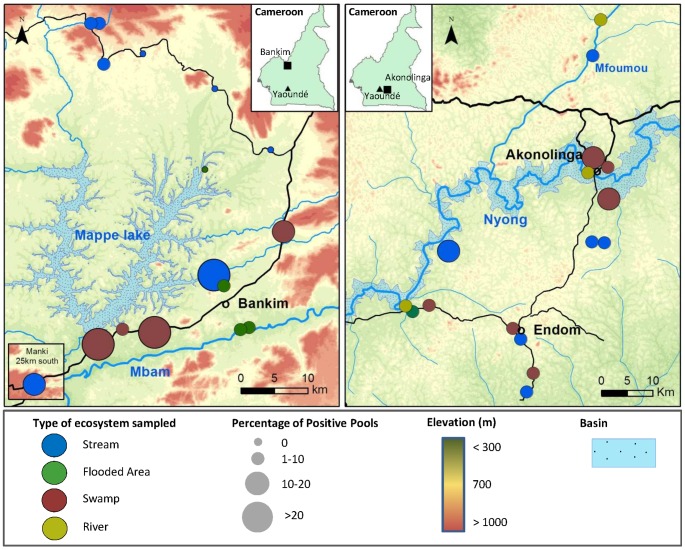
*M. ulcerans* spatial distribution in water bodies sampled in Cameroon from June 2012 to Mai 2013. Maps show regional distribution of *M. ulcerans* in water bodies sampled in Bankim (Left) and Akonolinga (Right). Each circle is a site and colors represent the type of ecosystem sampled. The size of the circles varies according to the percentage of pools that were qPCR positive to both KR and IS2404. Inlet figures illustrate a map of Cameroon with the location of Yaoundé, the capital city (dark triangle) and locations of Bankim and Akonolinga (dark squares).

*MU* was broadly distributed within both regions, and was found at least once in more than 80% of sites sampled during the year, with different distribution patterns for each region (Figure 1). In Akonolinga, *MU* was detected in all sites at least once during the year regardless of the geographical location or the type of ecosystem sampled. *MU* distribution in Bankim was more restricted, with 4 out of 16 sites found negative all year long, notably from streams in the northern part of the region. Overall, the proportion of positive sample-pools (hereafter defined as “pool positivity” or “pool prevalence”) ranged from 0 to 25% in the different sites, with the highest rates distributed along the road in the southern part of Bankim between the Mapé Dam and the Mbam River, and close to the basin of the Nyong river in Akonolinga (in swamps and streams nearby).

**Figure 2:**
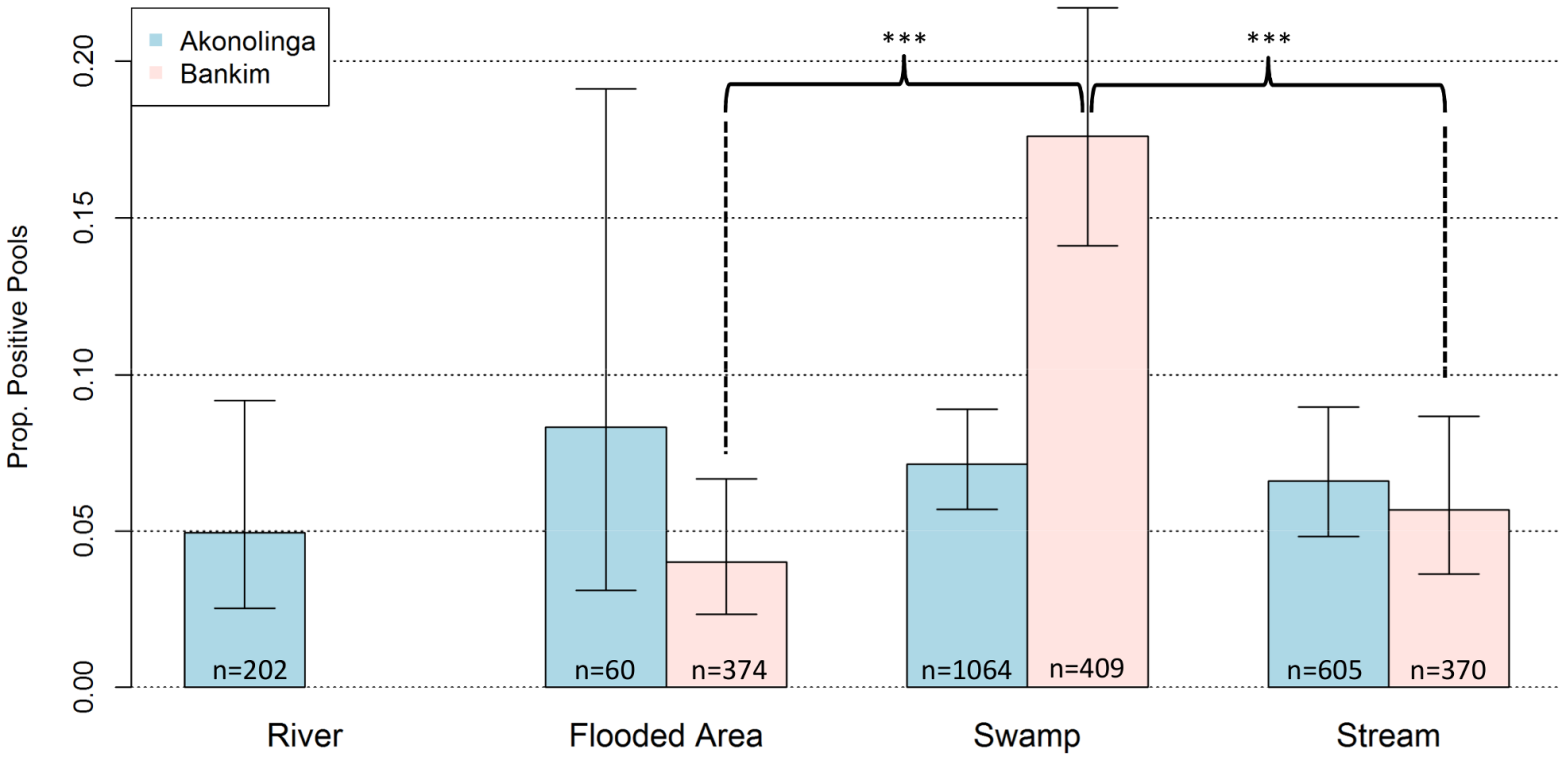
Overall distribution of *M.ulcerans* in aquatic ecosystems. Bars represent total proportion of *M. ulcerans* DNA positive sample-pools from each type of ecosystem in Akonolinga (blue) and Bankim (red). Whiskers indicate 95% Confidence intervals for the proportions. Asterisks represent significant differences in positivity between ecosystems within each region (χ^2^ test, p-value<0.0001).

Aquatic ecosystems with stagnant waters appeared to be associated with higher *MU* presence (Figure 2). We found *MU* in aquatic organisms from all four types of aquatic ecosystems sampled, with an average of 7.7% of positive sample-pools across ecosystems. Overall, positivity rate was 4.9% in rivers, 4.6% in flooded areas, 10.0% in swamps and 6.2% in streams. We found that swamps had significantly higher positivity than all other ecosystems in Bankim, with positivity in swamps 3 and 5 times higher than in streams and flooded areas respectively (*χ* ^2^ test, *p*-value <0.0001 for both). However, no significant differences in *MU* presence were found for any given environment in Akonolinga, although positivity in flooded areas and swamps was slightly higher.

#### Distribution of *M. ulcerans* in the aquatic community

A total number of 238,496 individuals were collected and classified over the course of the study, 200,918 in Akonolinga and 37,578 in Bankim. According to the pooling strategy described above, 145,255 of those (61%) were distributed in 3,084 sample-pools and analyzed by qPCR. 65 distinctive taxa were identified (Table S1). 85% of the whole aquatic community overall was made up of only 5 taxonomic orders: Coleoptera, Diptera, Ephemeroptera, Odonata and Hemiptera (Table 1). Among these, the most abundant families were Baetidae (18%), Noteridae (12%), Chironomidae (11%) and Hydrophilidae (7%). Aquatic vertebrates (fishes, tadpoles) and semi-aquatic or terrestrial orders (Araneae, Lepidoptera larvae, Collembola) represented 4% and 2%, respectively. Among aquatic ecosystems, water bodies with standing and slow flowing waters had less biodiversity in terms of number of orders, and were dominated by the 5 most abundant orders mentioned above (Table S2). Conversely, streams had higher biodiversity, with a larger proportion of other groups such as Decapoda and Trichoptera.

*MU* was present in nearly all taxonomic groups of the aquatic community and it was approximately evenly distributed among the whole aquatic community (Table 2). Pool prevalence for most of the groups was between 5–15%. Larvae of the order Lepidoptera had the highest pool prevalence overall (13.6%), followed by Annelida (12.3%) and Hemiptera (11.4%). However, regional differences in *MU* distribution should be noted: most of positive Lepidoptera and Annelida came from Bankim, where positivity was nearly 3 times higher for both groups than in Akonolinga (20.8 and 17.7% in Bankim compared to 5.0 and 6.7% in Akonolinga, respectively), although these differences were not significant. The lowest pool prevalence among positive groups was found in Acari (2.8%), Mollusca (3.3%) and Araneae (5.6%). Finally, we failed to detect *MU* only in two taxonomic groups: Trichoptera (89 pools tested, 1,434 individuals) and Collembola (28 pools tested, 79 individuals).

**Table 2:** Overall abundance and *M. ulcerans* presence in pools of aquatic vertebrates and macro-invertebrates.

			Akonolinga		Bankim		Total	
			Abundance (%)	KR+ & IS24+/Total (%)	Abundance (%)	KR+ & IS24+/Total (%)	Abundance (%)	KR+ & IS24+/Total (%)
**Vertebrates**	Fish		1101(0.55)	6/66 (9.1)	469(1.26)	5/57 (8.8)	1570(0.66)	11/123(8.9)
	Anura		5816(2.9)	6/102 (5.9)	1423(3.84)	4/47 (8.5)	7239(3.04)	10/149(6.7)
**Invertebrates**	Insecta	Odonata	23515(11.72)	18/242 (7.4)	5822(15.7)	16/120 (13.3)	29337(12.34)	34/362(9.4)
		Ephemeroptera	43874(21.86)	12/239 (5)	5409(14.58)	14/118 (11.9)	49283(20.72)	26/357(7.3)
		Hemiptera	17319(8.63)	32/263 (12.2)	3129(8.44)	13/133 (9.8)	20448(8.6)	45/396(11.4)
		Coleoptera	56351(28.08)	13/322 (4.0)	5343(14.4)	17/191 (8.9)	61694(25.94)	30/513(5.8)
		Diptera	30979(15.43)	24/269 (8.9)	9993(26.94)	15/144 (10.4)	40972(17.23)	39/413(9.4)
		Trichoptera	2958(1.47)	0/70 (0)	290(0.78)	0/19 (0)	3248(1.37)	0/89(0)
		Plecoptera	28(0.01)	0/0	4(0.01)	1/2 (50)	32(0.01)	1/2(50)
		Lepidoptera	372(0.19)	1/20 (5)	126(0.34)	5/24 (20.8)	498(0.21)	6/44(13.6)
	Mollusca		3022(1.5)	4/67 (6)	2712(7.31)	1/83 (1.2)	5734(2.41)	5/150(3.3)
	Crustacea	Decapoda	7763(3.87)	3/45 (6.7)	195(0.52)	0/2 (0)	7958(3.35)	3/47(6.4)
		Cladocera	947(0.47)	0/10 (0)	279(0.75)	2/11 (18.2)	1226(0.52)	2/21(9.5)
	Annelida		1715(0.86)	4/60 (6.7)	649(1.75)	11/62 (17.7)	2364(1)	15/122(12.3)
	Arachnida	Acari	1780(0.89)	3/59 (5.1)	274(0.74)	0/48 (0)	2054(0.86)	3/107(2.8)
		Araneae	2655(1.32)	5/81 (6.2)	868(2.34)	4/79 (5.1)	3523(1.48)	9/160(5.6)
	Collembola		514(0.26)	0/16 (0)	107(0.29)	0/12 (0)	621(0.26)	0/28(0)
	**Total**		**200709 (100)**	**126/1931 (6.5)**	**37092 (100)**	**108/1152 (9.4)**	**237801 (100)**	**239/3084 (7.7)**

Results are given for Akonolinga (12 months of sampling) and Bankim (4 months of sampling). Abundance indicates total number of individual organisms collected of each taxonomic group. *M. ulcerans* presence was assessed by qPCR (KR and IS2404). Only sample-pools positive to both sequences were considered positive.

### Ecological dynamics along the year

#### Monthly fluctuations of *M. ulcerans* presence in aquatic ecosystems

**Figure 3:**
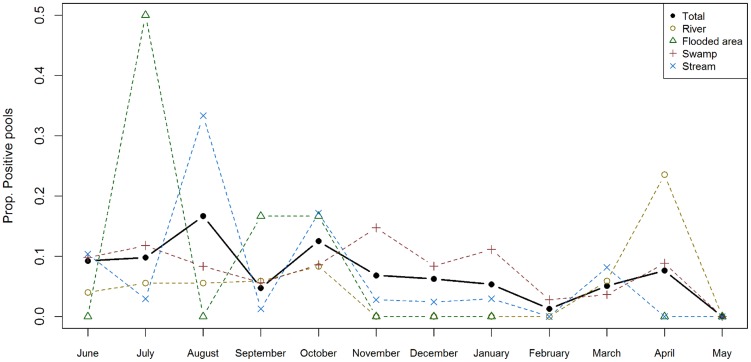
Monthly distribution of *M. ulcerans* positivity rate in sample-pools from aquatic ecosystems in Akonolinga from June 2012 to May 2013. Values indicate the proportion of pools of aquatic organisms collected from a specific ecosystem that were positive to *M.ulcerans* at a given month. The solid line in black represents the total trend (all ecosystems); dashed lines represent trends for pools from each type of ecosystem. Missing information for flooded areas in February and March is due to lack of water in those sites, which prevented sampling.

*MU* was present in aquatic ecosystems nearly all year long. In Akonolinga, where samples were collected every month, *MU* was only absent in May, and in Bankim we detected *MU* in all four time steps sampled (every three months). In this section, only the dynamics for the 12 months in Akonolinga are shown (Figures S1 and S2 show the dynamics in Bankim). *MU* presence fluctuated through time (Figure 3), with changes from 0 to 15% in total pool positivity. The largest peak in pool positivity was found in August and October, and we found a progressive drop in pool positivity from October to February.

**Figure 4:**
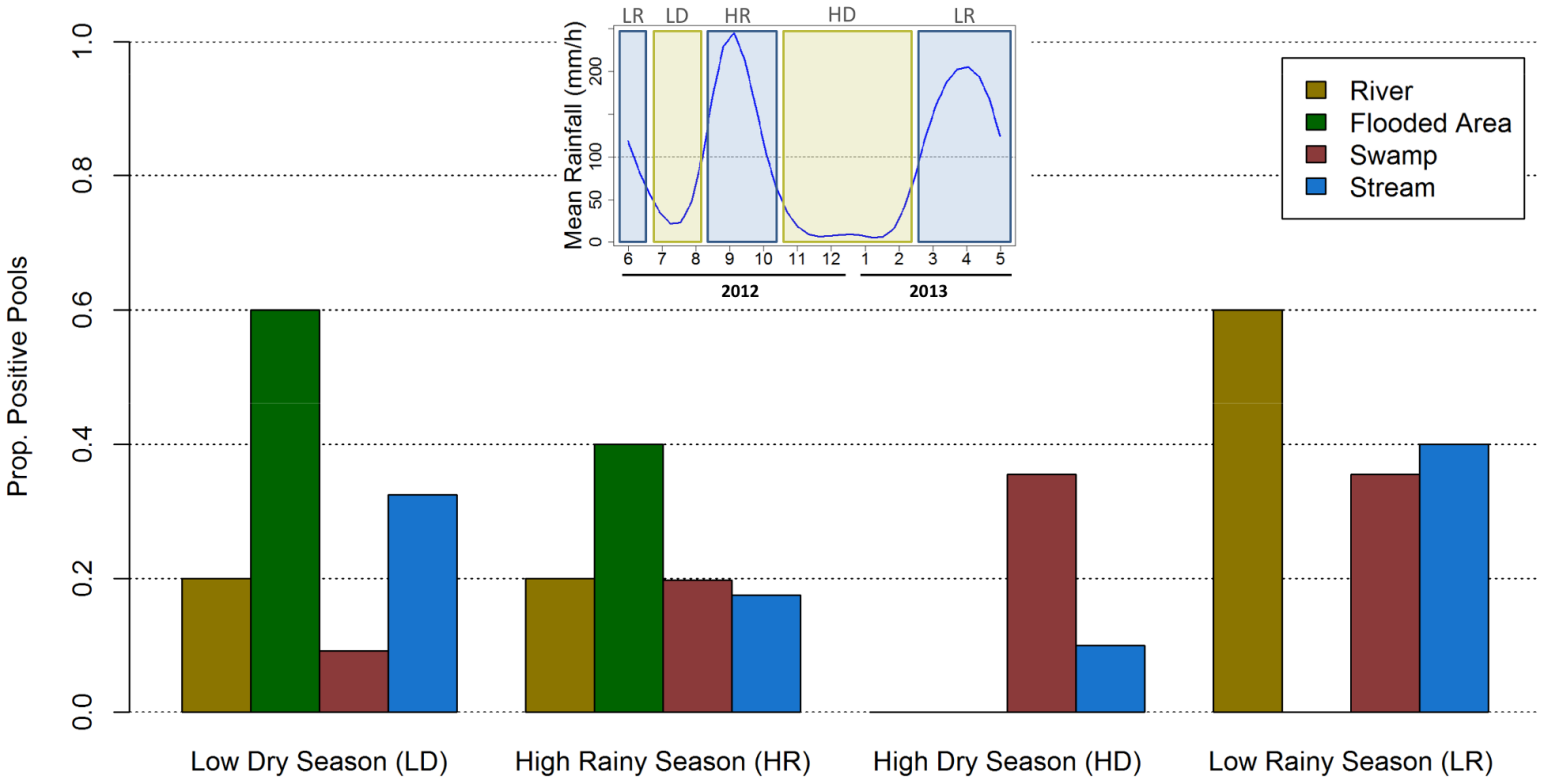
Seasonal distribution of *M. ulcerans* positive sample-pools from each type of ecosystem. Inset figure on top indicates the rainfall patterns in Akonolinga from June 2012 to May 2013 and the cutting of the period sampled into two dry seasons (LD and HD; Rainfall <100 mm) and two rainy seasons (LR and HR; Rainfall >100 mm). Bars indicate the proportion of *M. ulcerans* positive sample-pools from a given season and ecosystem out of the total number of positive sample-pools from that ecosystem.

Each ecosystem had distinct temporal variations and a favorable time of the year for *MU* presence (Figure 3). In rivers and flooded areas, *MU* was absent for a long period of time (4 and 8 months respectively) and then experienced a sudden increase in pool positivity (in April and July respectively). As a result, more than half of positive sample-pools in these ecosystems were found in a specific season, the low rainy season for rivers and the low dry season for flooded areas (Figure 4). In swamps and streams, the seasonal effect was less pronounced with presence of *MU* most of the year and fluctuations in pool positivity that ranged from 0 to 15% for swamps and to 30% in streams. Over one third of positive sample-pools in swamps and streams were found during the low rainy season and around one third were found in another season (high dry season for swamps and low dry season for streams). Of all ecosystems, only *MU* positivity dynamics for rivers were correlated to rainfall dynamics (Figure S5).

#### Temporal dynamics of *M. ulcerans* presence in the aquatic community

**Figure 5:**
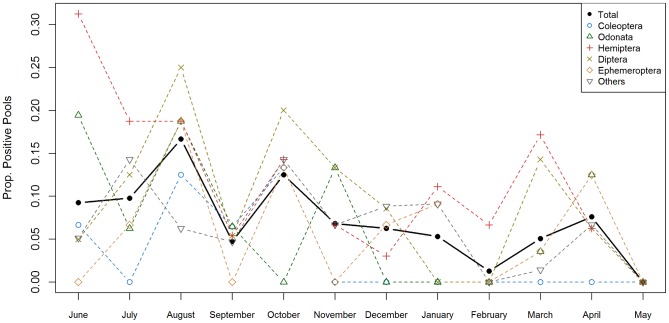
Monthly distribution of *M. ulcerans* positivity rate in pools of aquatic organisms in Akonolinga from June 2012 to May 2013. Values indicate the proportion of pools of aquatic organisms belonging to a specific taxon that were positive to *M.ulcerans* at a given month. Only the 5 most abundant taxonomic orders were systematically tested for all sites and months. The positivity dynamics for the rest of sample-pools are grouped as “others”. The solid line in black represents the total trend (all taxonomic groups); dashed lines represent trends for each taxonomic group.

*MU* colonization dynamics for the different taxonomic groups were highly variable (Figure 5). Hemipterans were the only group positive during 11 months of the year, whereas the order Coleoptera was repeatedly negative for more than half a year (from November to May). The highest peaks in pool positivity at any given month were for Hemiptera in June (>30%) and for Diptera in August (25%). Pool positivity in other orders was lower than 20% all year long. Out of the 5 orders systematically tested for all sites and months for *MU* presence, none of their colonization dynamics were correlated to rainfall (Figure S6).

## Discussion

Despite the great health and socio-economic burden borne by people affected with BU, little is known about the ecology and mode of transmission of this disease. *MU* is embedded in an environment that is inherently dynamic, but information on spatio-temporal dynamics of *MU* persistence and spread is dramatically lacking. The results shown here represent a step forward in the understanding of MU ecology. They provide the first account of *MU* spatio-temporal dynamics in aquatic communities from a variety of ecosystems within BU endemic regions. We show first that *MU* is ubiquitous within these regions and can be found in all types of freshwater ecosystems, but swampy areas seem more favorable to MU presence, as demonstrated in Bankim. Then, we confirm that *MU* is present in nearly all taxonomic groups of the aquatic community, but we show that groups common in streams are minimally colonized. Finally, we demonstrate that *MU* has distinctive temporal dynamics in each ecosystem and taxonomic group, suggesting that *MU* occurrence is probably driven by complex ecological interactions between environmental abiotic and biotic factors.

We found that *MU* presence in Bankim was more restricted to the south of this area, especially between the Mapé Dam and the Mbam River, where BU cases concentrate [57] and more swamps and flooded areas prevail. The construction of the dam has been previously associated to the emergence of cases in the area [8], [22] and proximity to the Mbam River was found to be a risk factor in a case-control study [22]. However, our results suggest that swamps created along the road, rather than the flooded areas created artificially by the dam or naturally near the Mbam after heavy rains, are more favorable to the presence of *MU*. Swamps are characterized by stagnant waters with low oxygen and high temperatures, which may create conditions favorable to *MU* growth and specific fauna in which to develop [16]–[19]. Furthermore, while water level and conditions in flooded areas are highly variable throughout the year, swamps are more stable environments, which could influence the differences observed in these two stagnant ecosystems [58].

In contrast, *MU* is present everywhere across the Akonolinga region and all environments presented very similar positivity, although the highest positivity concentrated near the basin of the Nyong river. While climate, land cover or human modifications of the environment could be behind these disparate regional distributions, it could also reflect a spread of the bacteria over time. Indeed, it is possible that *MU* initially persists in the most favorable environments (swamps), as in the case of Bankim where cases have been reported for less than 10 years [8], spreading over time to other environments where water conditions and aquatic communities are less favorable and/or intermittent along the year, as in the case of Akonolinga where *MU* is endemic for more than 40 years [6]. Flying insects could be responsible of this dissemination as previously suggested [38], [59]. Out of the two taxonomic orders that are both aquatic in adult stage and capable of flight (Coleoptera and Hemiptera), only Hemiptera was found positive in all types of ecosystem. Indeed, this group was found positive to *MU* in 65% of the sites, more than any other group of the aquatic community (table S4).

*MU* is present in nearly every group of the aquatic community and no taxonomic group stands among others as the major host carrier of *MU*. Aquatic vertebrates and invertebrates, as well as semi-aquatic groups, are positive for IS2404 and KR, with similar pool prevalence. This is in line with the idea of multi-host transmission dynamics and more particularly a transmission through ecological webs, where some species can highly contribute to *MU* transmission without experiencing a significantly larger positivity [32]. Nevertheless, some patterns arise for several specific taxonomic groups. Firstly, the most positive order in terms of pool prevalence are Lepidoptera larvae (caterpillars), an invertebrate with semi-aquatic families mostly living and feeding on riverine aquatic plants [42]. This finding suggests that some aquatic plants might play an important role on *MU* persistence and development in the aquatic ecosystem or in ecotone areas, and be a source of infection for herbivorous invertebrates. Indeed, some plants could harbor *MU* in endemic regions [24], [60] and they stimulate its growth under experimental conditions [61]. Secondly, groups of aquatic invertebrates that were found mainly in streams such as Trichoptera and Decapoda are among the groups with the lowest pool prevalence. These findings support the hypothesis that *MU* might not be well adapted to environmental conditions in this type of aquatic ecosystems.

Regarding the seasonal dynamics, *MU* is present in freshwater ecosystems and aquatic organisms throughout the year but there are fluctuations both between seasons and within each season, as previously demonstrated for *MU* colonization of water bugs [38]. The highest peak in positivity appears in August and October (*i.e.* over the high rainy season), and then decreases progressively throughout the high dry season (November to February). These findings could be consistent with the idea of a run-off of bacteria into the aquatic environment during periods of intensive rainfall, as previously suggested [24], [62]. However, the lack of correlation between rainfall patterns and the dynamics observed for the various ecosystems and taxonomic orders highlights that more complex interactions might take place within the aquatic community. Differences in feeding habits may explain the distinct colonization dynamics of different orders. For instance, while Hemiptera were found positive all year long (except in May), Coleoptera were repeatedly found negative for more than half a year (Figure 5). These two orders share many common features: they have both larval and adult aquatic stages, many are capable of flight, and their abundance dynamics along the year are very similar (Figure S3 and S4). However, while most families of Hemiptera are voracious predators of aquatic organisms (only Corixidae feed on aquatic plants), families of Coleoptera present a large spectrum of feeding habits that include predators, shredders, scrappers, filtering collectors and omnivorous organisms [41], [42]. Laboratory experiments support the idea of a trophic transmission of *MU* through predation [30], [34], [63], [64] and a mathematical model studying *MU* prevalence within 27 aquatic communities in Ghana suggested that a transmission through ecological webs is more likely than a purely environmental acquisition from contaminated water [32]. Our results support the hypothesis that biotic interactions may play a role in *MU* transmission and that *MU* dynamics could result from a complex interplay between environmental abiotic factors and variations in community assemblages.

We show that important fluctuations in *MU* positivity take place within each particular ecosystem. For most sites, we checked for the presence of *MU* in a given month and site by analysing 6 pools of aquatic organisms. This may be insufficient to demonstrate the absence of the bacteria in the ecosystem, since pool positivity overall was lower than 10%. We attempted to increase the chances to detect *MU* by pooling all individuals of the most abundant taxonomic orders in the aquatic ecosystem, which allowed us to pool and analyze over 60% of the 238,496 individuals sampled without losing comparability of the results. Furthermore, disparate sampling strategies for each region could be behind the differences found between the types of environment for the two study regions. Bankim was only sampled 4 months of the year as opposed to 12 months in Akonolinga. Therefore, we cannot rule out the possibility that sampling in Bankim may have taken place at appropriate times of the year for swamps but not for the other environments in this region. We tried to avoid this by sampling in Bankim at regular intervals (every three months), therefore capturing a maximum of variability along the year.

This study reinforces the idea that *MU* persists in a wide range of locations [24], [40] and taxonomic groups [28] and the pool positivity rates described here (nearly 10% overall) are consistent with previous studies [8], [28], [38]. This ubiquity of *MU* and its persistence in the environment throughout the year contrast with the focal distribution and low number of BU human cases in endemic regions. A possible explanation is that while we are likely to be detecting one (or several) of the *MU* ecovars present in the environment (previously referred to as mycolactone producing mycobacteria), this does not necessarily imply that we are detecting strains of *MU* with pathogenic potential to cause BU in humans [17], [65]–[67]. Future studies comparing the strain diversity of environmental and human samples with molecular techniques such as SNP typing [68], [69] could shed some light on this issue. Furthermore, we rely as previous studies on qPCR amplification of KR and IS2404 sequences as an indicator of the presence of *MU*, which gives no certainty of whether the DNA detected belongs to viable mycobacteria. The lack of an appropriate technique to culture *MU* from the environment remains a major limitation of fieldwork studies. Nevertheless, qPCR remains the gold standard for environmental studies on *MU* ecology [8], [27], [38], [40]. An alternative hypothesis is that while the presence of *MU* in the environment reflects a potential risk for infection, many environmental and socio-economic factors may need to come together to enable *MU* transmission to humans. Sero-epidemiological studies have shown that a large proportion of the population living in endemic regions have been exposed to *MU*, but only a small fraction develop the disease [70]. Therefore, *MU* might only trigger BU disease under certain environmental conditions (a bacterial concentration threshold and/or contact with a competent, infected vector) or in subpopulations in high contact with potential sources of infection and with increased susceptibility to infection (due to immunity, hygiene, etc.).

In conclusion, this study provides for the first time a detailed characterization through space and time of *MU* presence in two BU endemic regions with distinct environmental conditions. The understanding of *MU* ecology to date is still limited, especially regarding the conditions that allow this mycobacterium to persist in the environment and be transmitted to humans. Our study attempts to complete previous approaches by sampling multiple aquatic communities over time in order to better understand the influence of aquatic ecosystems on *MU* presence and its dynamics along the year. The global trend we describe for *MU* dynamics could be the result of complex ecological processes, with interactions between environmental abiotic and biotic factors that require deeper analysis, something that is beyond the scope of this paper. However, we believe that coupling data produced by such field studies with fine-scale epidemiological data and integrated through statistical and mathematical models could provide a major step forward in the understanding of *MU* ecology and BU mode of transmission.

## Supporting Information

Figure S1 **Monthly distribution of** ***M. ulcerans*** **positivity rate in pools from aquatic ecosystems in Bankim from June 2012 to March 2013.** Values indicate the proportion of pools of aquatic organisms collected from a specific ecosystem that were positive to *M.ulcerans* at a given month. The solid line in black represents the total trend (all ecosystems); Dashed lines represent trends for pools from each type of ecosystem.
(TIF)

Figure S2 **Monthly distribution of** ***M. ulcerans*** **positivity rate in pools of aquatic organisms in Bankim from June 2012 to March 2013.** Values indicate the proportion of pools of aquatic organisms belonging to a specific taxon that were positive to *M.ulcerans* at a given month. Only the 5 most abundant taxonomic orders were systematically tested for all sites and months. The positivity dynamics for the rest of pools are grouped as “others”. The solid line in black represents the total trend (all taxonomic groups); Dashed lines represent trends for each taxonomic group.
(TIF)

Figure S3 **Abundance dynamics of aquatic organisms in Akonolinga from June 2012 to May 2013.** Abundance values are normalized within each group by dividing abundance for a given month by the maximal abundance for that group. The solid line in black represents the total trend (all taxonomic groups); Dashed lines represent trends for each taxonomic group. Only the 5 most abundant orders are represented. The rest of orders are grouped as “others.
(TIF)

Figure S4 **Abundance dynamics of aquatic organisms in Bankim from June 2012 to March 2013.** Abundance values are normalized within each group by dividing abundance for a given month by the maximal abundance for that group. The solid line in black represents the total trend (all taxonomic groups); Dashed lines represent trends for each taxonomic group. Only the 5 most abundant orders are represented. The rest of orders are grouped as “others.
(TIF)

Figure S5 **Temporal cross-correlation of monthly rainfall distribution and** ***M. ulcerans*** **positivity rate in pools from aquatic ecosystems in Akonolinga from June 2012 to May 2013.** Vertical bars indicate the strength of the correlation between the two series for a given lag (in months). Horizontal dashed blue lines represent the threshold of statistical significance.
(TIF)

Figure S6 **Temporal cross-correlation of monthly rainfall distribution and** ***M. ulcerans*** **positivity rates in pools of aquatic organisms in Akonolinga from June 2012 to May 2013.** Vertical bars indicate the strength of the correlation between the two series for a given lag (in months). Horizontal blue dashed lines represent the threshold of statistical significance.
(TIF)

Table S1 **Overall abundance of aquatic vertebrates and macro-invertebrates at the lowest classification level achieved.** Results are given for Akonolinga (12 months of sampling) and Bankim (4 months of sampling). Abundance indicates total number of individual organisms collected of each taxonomic group.
(PDF)

Table S2 **Total and relative abundance of aquatic vertebrates and macro-invertebrates in aquatic ecosystems.** Results are given for Akonolinga (12 months of sampling) and Bankim (4 months of sampling). Total abundance indicates total number of individual organisms collected of each taxonomic group in a given ecosystem. Relative abundance (in brackets) indicates the percentage of individuals from each ecosystem belonging to that taxonomic group.
(PDF)

Table S3 **Detection of** ***M. ulcerans*** **DNA by qPCR with KR and IS2404 sequences.** Results are given for pools of aquatic vertebrates and invertebrates from Akonolinga (12 months of sampling) and Bankim (4 months of sampling). All pools were tested for the KR sequence. KR positive pools where then confirmed by IS2404. Only sample-pools positive to both sequences were considered positive.
(PDF)

Table S4 **Distribution of** ***M. ulcerans*** **positive macro-invertebrates and vertebrates over space and time.** Values indicate the number of sites and months where a taxonomic group has been found positive to *M. ulcerans* DNA by both IS2404 and KR out of the total number of sites and months where the group has been tested.
(PDF)
