# Supplementary figures and images for: Mycobacterium ulcerans Ecological Dynamics and Its Association with Freshwater Ecosystems and Aquatic Communities: Results from a 12-Month Environmental Survey in Cameroon
Source: PLoS Negl Trop Dis. 2014 May 15;8(5):e2879. doi: 10.1371/journal.pntd.0002879 (PMC4022459; doi:10.1371/journal.pntd.0002879)

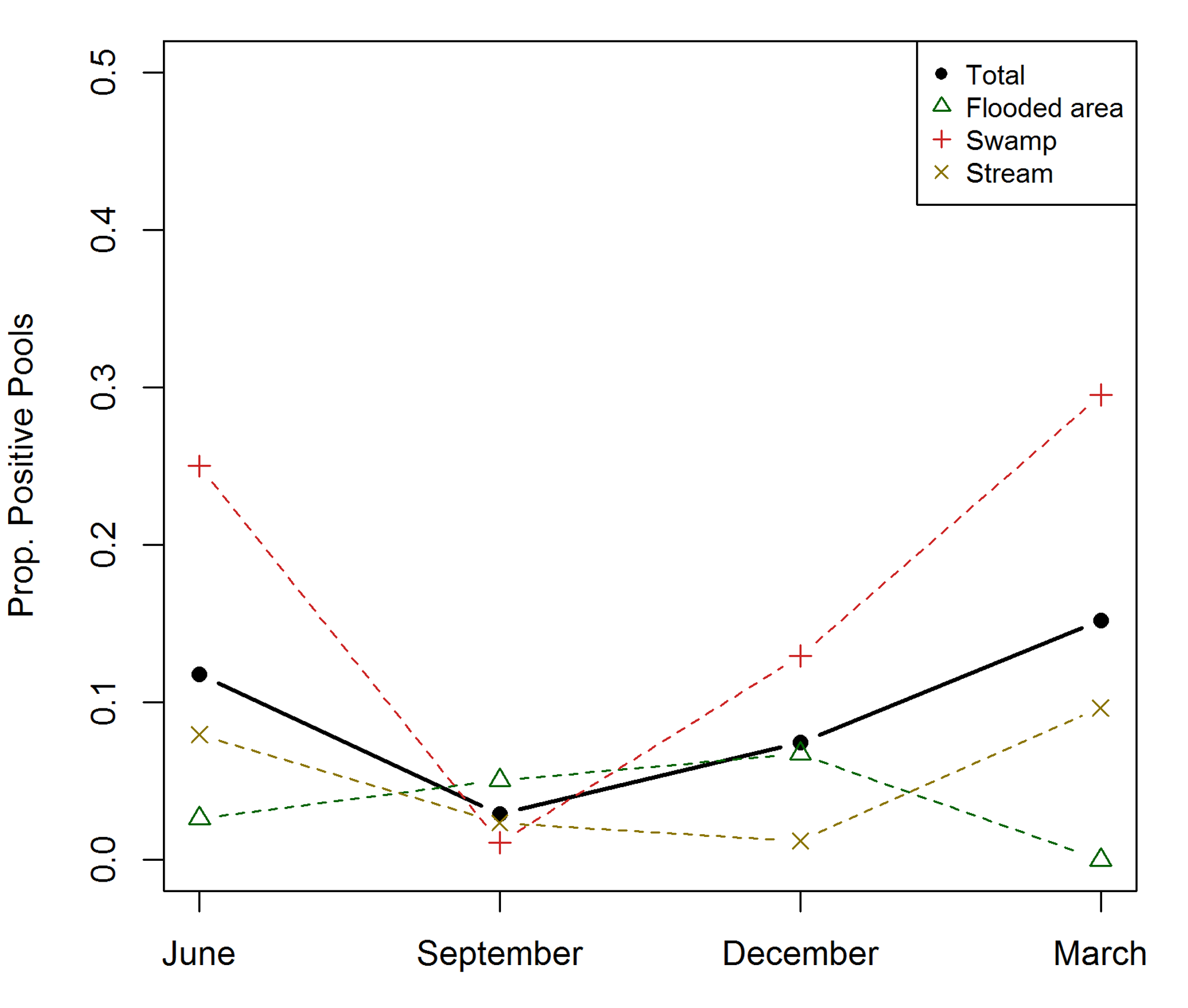

Supplement: Figure S1 — Monthly distribution of M. ulcerans positivity rate in pools from aquatic ecosystems in Bankim from June 2012 to March 2013. Values indicate the proportion of pools of aquatic organisms collected from a specific ecosystem that were positive to M.ulcerans at a given month. The solid line in black represents the total trend (all ecosystems); Dashed lines represent trends for pools from each type of ecosystem. (TIF) [file pntd.0002879.s001.tif]

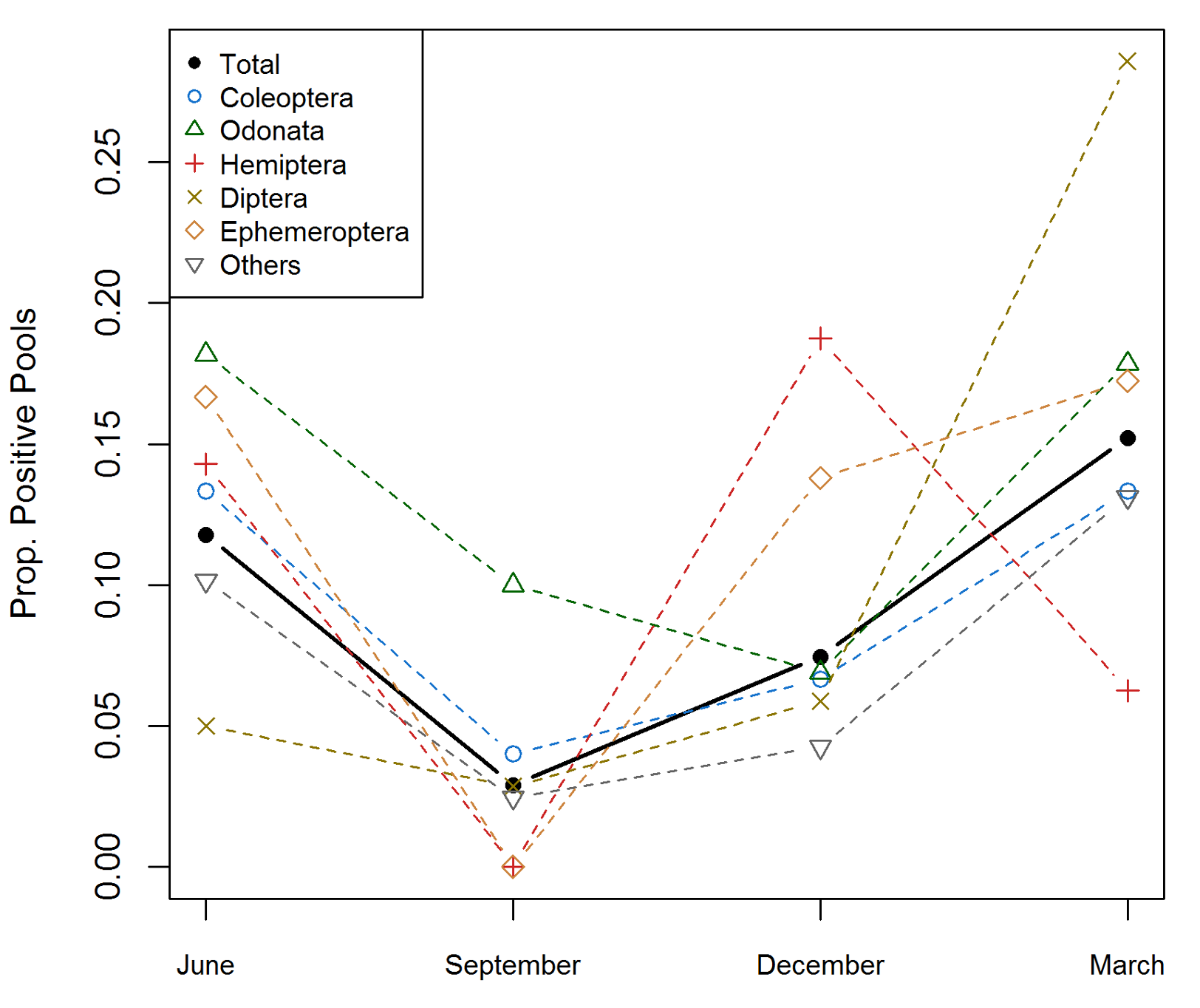

Supplement: Figure S2 — Monthly distribution of M. ulcerans positivity rate in pools of aquatic organisms in Bankim from June 2012 to March 2013. Values indicate the proportion of pools of aquatic organisms belonging to a specific taxon that were positive to M.ulcerans at a given month. Only the 5 most abundant taxonomic orders were systematically tested for all sites and months. The positivity dynamics for the rest of pools are grouped as “others”. The solid line in black represents the total trend (all taxonomic groups); Dashed lines represent trends for each taxonomic group. (TIF) [file pntd.0002879.s002.tif]

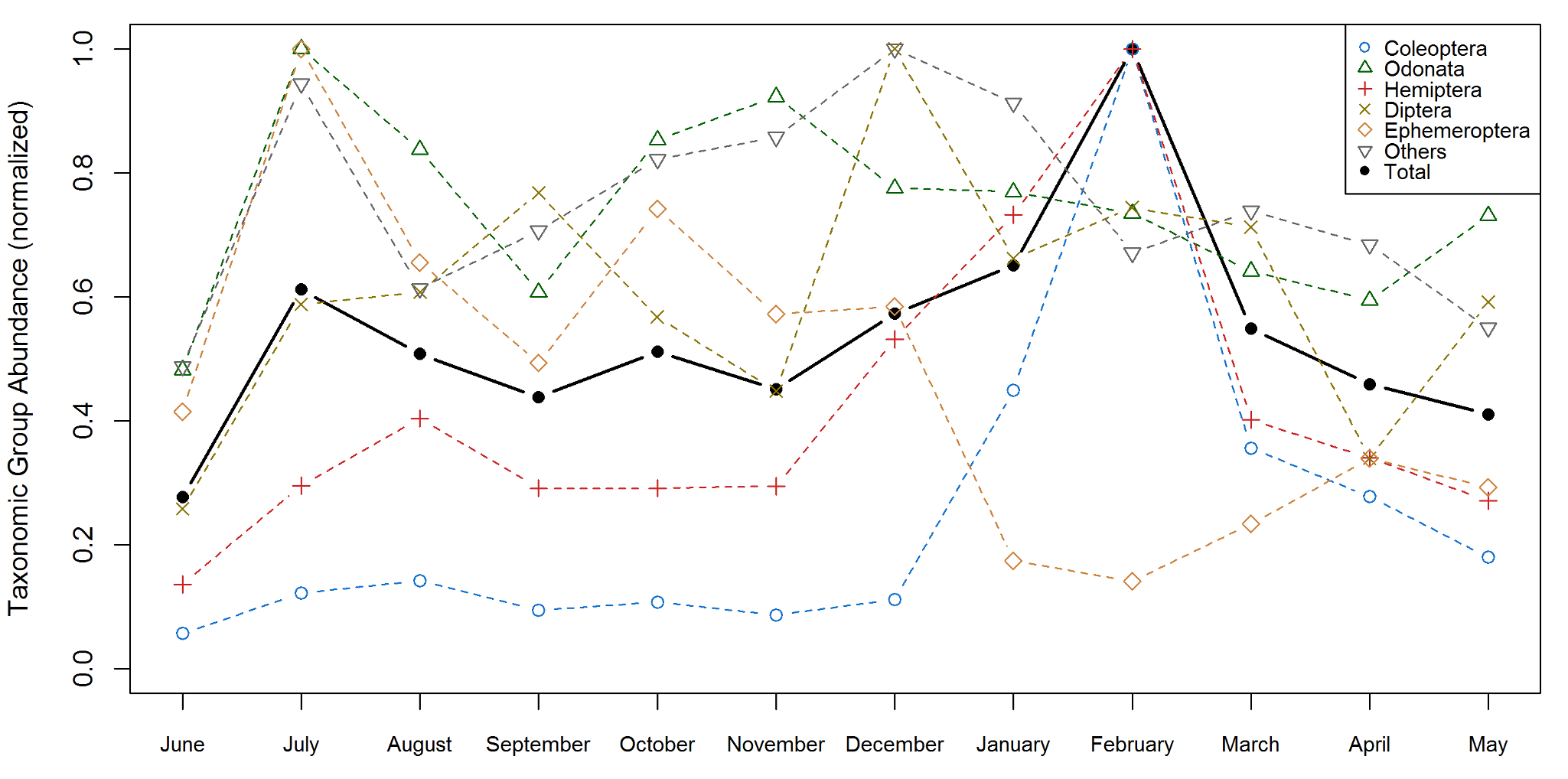

Supplement: Figure S3 — Abundance dynamics of aquatic organisms in Akonolinga from June 2012 to May 2013. Abundance values are normalized within each group by dividing abundance for a given month by the maximal abundance for that group. The solid line in black represents the total trend (all taxonomic groups); Dashed lines represent trends for each taxonomic group. Only the 5 most abundant orders are represented. The rest of orders are grouped as “others. (TIF) [file pntd.0002879.s003.tif]

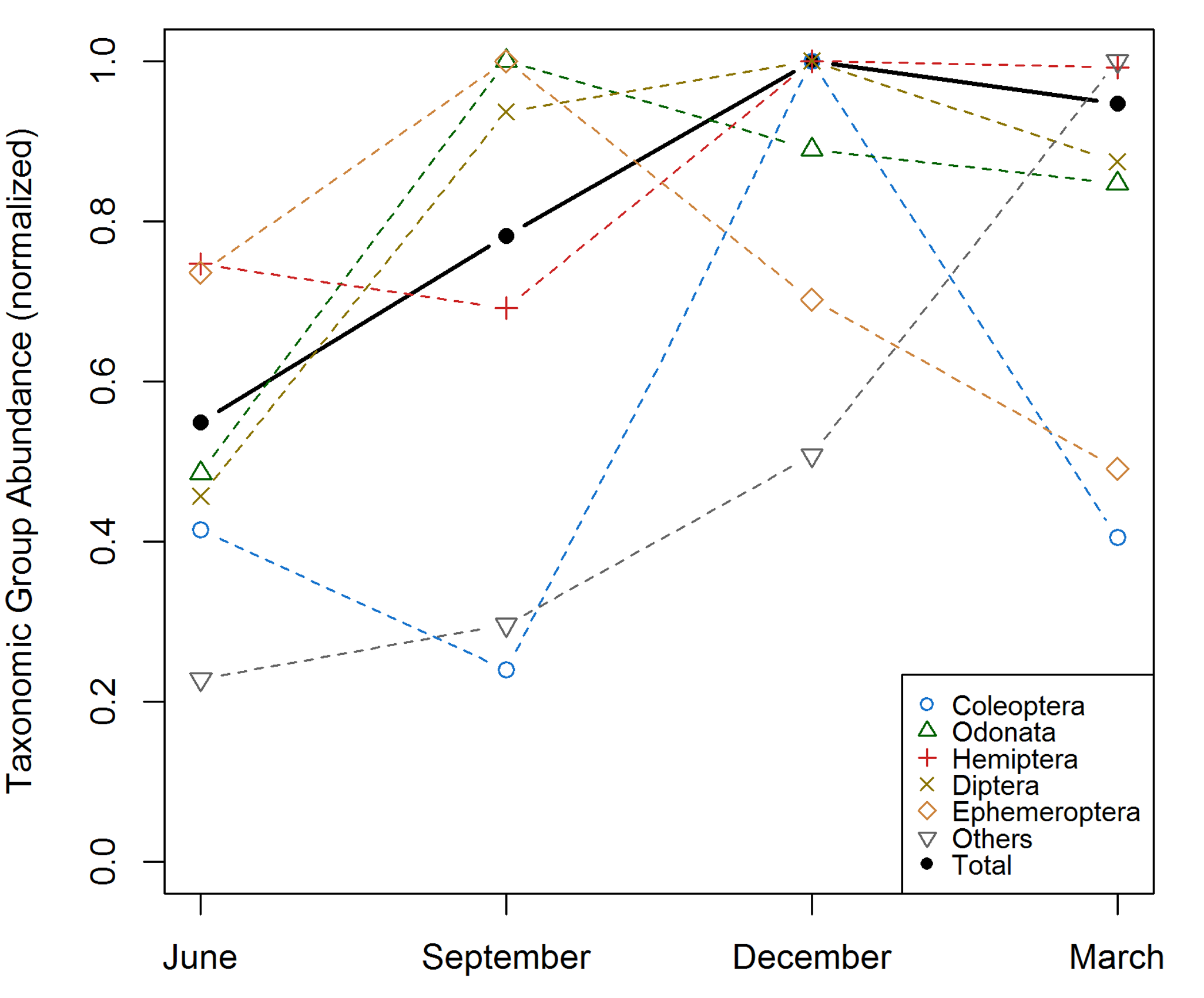

Supplement: Figure S4 — Abundance dynamics of aquatic organisms in Bankim from June 2012 to March 2013. Abundance values are normalized within each group by dividing abundance for a given month by the maximal abundance for that group. The solid line in black represents the total trend (all taxonomic groups); Dashed lines represent trends for each taxonomic group. Only the 5 most abundant orders are represented. The rest of orders are grouped as “others. (TIF) [file pntd.0002879.s004.tif]

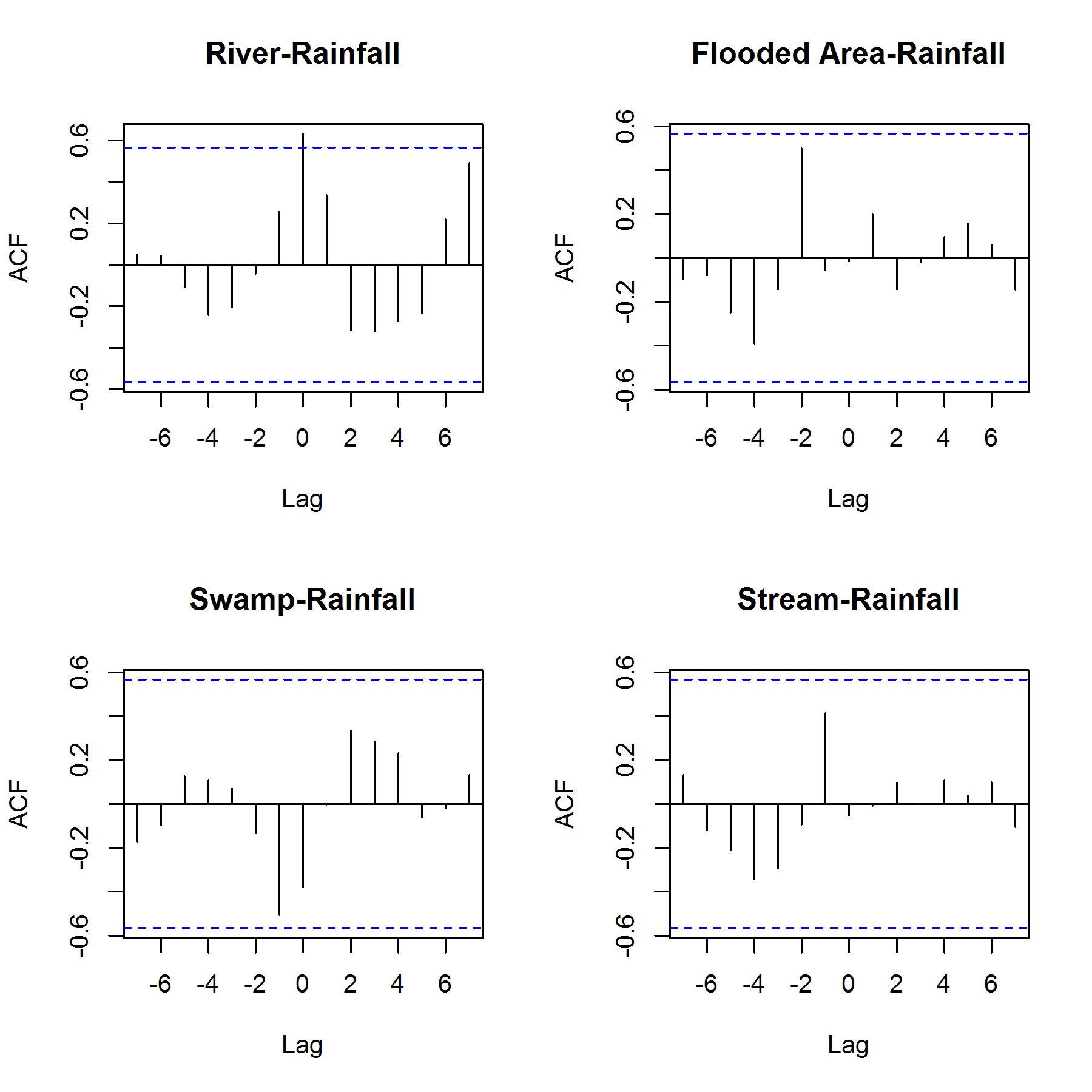

Supplement: Figure S5 — Temporal cross-correlation of monthly rainfall distribution and M. ulcerans positivity rate in pools from aquatic ecosystems in Akonolinga from June 2012 to May 2013. Vertical bars indicate the strength of the correlation between the two series for a given lag (in months). Horizontal dashed blue lines represent the threshold of statistical significance. (TIF) [file pntd.0002879.s005.tif]

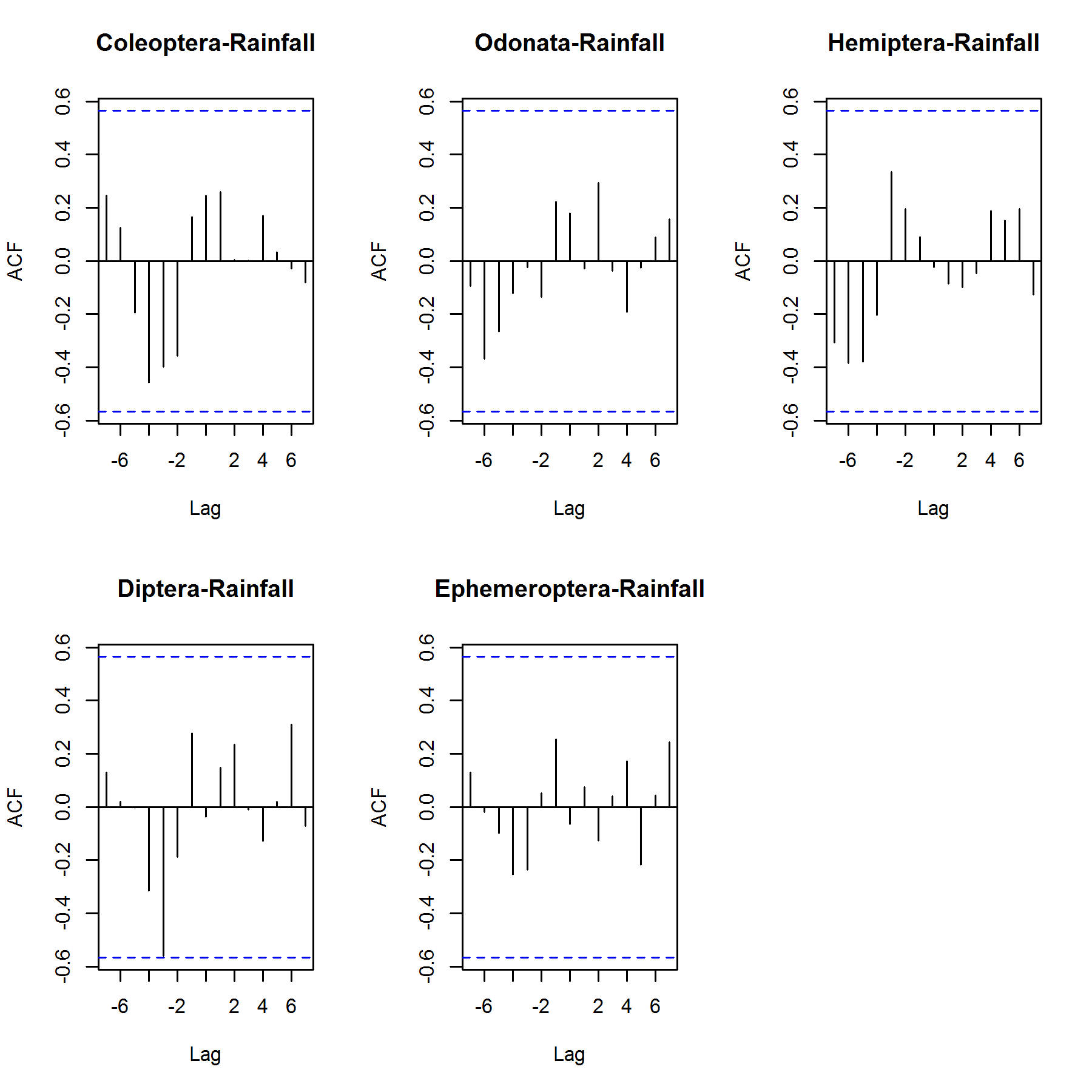

Supplement: Figure S6 — Temporal cross-correlation of monthly rainfall distribution and M. ulcerans positivity rates in pools of aquatic organisms in Akonolinga from June 2012 to May 2013. Vertical bars indicate the strength of the correlation between the two series for a given lag (in months). Horizontal blue dashed lines represent the threshold of statistical significance. (TIF) [file pntd.0002879.s006.tif]
