# Supplementary material for: Mycobacterium ulcerans Ecological Dynamics and Its Association with Freshwater Ecosystems and Aquatic Communities: Results from a 12-Month Environmental Survey in Cameroon
Source: PLoS Negl Trop Dis. 2014 May 15;8(5):e2879. doi: 10.1371/journal.pntd.0002879 (PMC4022459; doi:10.1371/journal.pntd.0002879)
Supplement: Table S1 — Overall abundance of aquatic vertebrates and macro-invertebrates at the lowest classification level achieved. Results are given for Akonolinga (12 months of sampling) and Bankim (4 months of sampling). Abundance indicates total number of individual organisms collected of each taxonomic group. (PDF) [file pntd.0002879.s007.pdf]

Table S1. Overall abundance of aquatic vertebrates and macro-invertebrates at the lowest classification level achieved. Results are given for Akonolinga (12 months of sampling) and Bankim (4 months of sampling). Abundance indicates total number of individual organisms collected of each taxononomic group.

	Higher Classification	Order	Stage	Family	Akonolinga	Bankim	Total
**Vertebrates**	Fish		Adult		600	289	889
			Fry		501	180	681
	Anura		Adult		14	0	14
			Tadpole		5802	1423	7225
**Invertebrates**	Insecta	Odonata	Larvae	Anisoptera*	13013	1934	14947
				Zygoptera*	10502	3888	14390
		Ephemeroptera	Larvae	Baetidae	38335	4668	43003
				Caenidae	3022	466	3488
				Heptageniidae	1336	89	1425
				Leptophlebidae	1181	186	1367
		Hemiptera	Adult	Belostomatidae	1547	156	1703
				Naucoridae	237	76	313
				Hydrometridae	490	76	566
				Nepidae	730	311	1041
				Corixidae	550	221	771
				Notonectidae	2490	805	3295
				Pleidae	6996	150	7146
				Gerridae	573	307	880
				Mesoveliidae	1187	460	1647
				Saldidae	74	12	86
				Veliidae	186	93	279
				Hebridae	9	1	10
				Leptopodidae	37	2	39
				Unknown	0	12	12
		Hemiptera	Larvae	Belostomatidae	2059	410	2469
				Naucoridae	85	32	117
				Others	69	5	74
		Coleoptera	Adult	Dytiscidae	6879	583	7462
				Hydrophilidae	15355	464	15819
				Curculionidae	56	5	61
				Gyrinidae	42	30	72
				Noteridae	26740	2563	29303
				Staphilinidae	171	34	205
				Chrisomelidae	54	22	76
				Haliplidae	184	7	191
				Lampiridae	0	3	3
				Carabidae	172	13	185
				Elmidae	186	40	226
				Dryopidae	21	9	30
				Hydrochidae	43	21	64
				Spercheidae	28	1	29
				Torridicolidae	6	1	7

Table S1. (cont.)

	Higher Classification	Order	Stage	Family	Akonolinga	Bankim	Total
**Invertebrates**	Insecta	Coleoptera	Adult	Hydraenidae	2	0	2
				Unknown	233	38	271
			Larvae	Scirtidae	874	134	1008
				Dityscidae	1481	218	1699
				Haliplidae	152	4	156
				Lampiridae	11	1	12
				Unknown (with jaws)	2019	737	2756
				Unknown (without jaws)	1642	415	2057
		Diptera	Larvae	Chironomidae	20626	6757	27383
				Culicidae	4360	599	4959
				Ceratopogonidae	1970	1463	3433
				Tipulidae	153	45	198
				Tabanidae	86	10	96
				Chaoboridae	1136	54	1190
				Empididae	14	7	21
				Simulidae	80	55	135
				Syrphidae	17	28	45
				Ephydridae	5	2	7
				Psychodidae	45	1	46
				Dolichopodidae	12	2	14
				Dixidae	3	4	7
				Muscidae	16	2	18
				Sciomyzidae	3	3	6
				Unknown	119	19	138
			Pupae		2334	942	3276
		Trichoptera	Larvae		2958	290	3248
		Plecoptera	Larvae		28	4	32
		Lepidoptera	Larvae		372	126	498
	Mollusca	Gastropoda			1410	1700	3110
		Bivalvia			1612	1012	2624
	Crustacea	Decapoda		Dendrobranchiata	7748	191	7939
				Pleocyemata	15	4	19
		Cladocera			947	279	1226
	Annelida	Hirudinea**			373	97	470
		Oligochaeta**			1342	552	1894
	Arachnida	Acari		Hydracarina	1780	274	2054
		Araneae			2655	868	3523
	Collembola				514	107	621
	**Total**				**200709**	**37092**	**237801**

* Suborder; **Subclass
