# Supplementary material for: Mycobacterium ulcerans Ecological Dynamics and Its Association with Freshwater Ecosystems and Aquatic Communities: Results from a 12-Month Environmental Survey in Cameroon
Source: PLoS Negl Trop Dis. 2014 May 15;8(5):e2879. doi: 10.1371/journal.pntd.0002879 (PMC4022459; doi:10.1371/journal.pntd.0002879)
Supplement: Table S2 — Total and relative abundance of aquatic vertebrates and macro-invertebrates in aquatic ecosystems. Results are given for Akonolinga (12 months of sampling) and Bankim (4 months of sampling). Total abundance indicates total number of individual organisms collected of each taxonomic group in a given ecosystem. Relative abundance (in brackets) indicates the percentage of individuals from each ecosystem belonging to that taxonomic group. (PDF) [file pntd.0002879.s008.pdf]

Table S2. Total and relative abundance of aquatic vertebrates and macro-invertebrates in aquatic ecosystems. Results are given for Akonolinga (12 months of sampling) and Bankim (4 months of sampling). Total abundance indicates total number of individual organisms collected of each taxonomic group in a given ecosystem. Relative abundance (in brakets) indicates the percentage of individuals from each ecosystem belonging to that taxonomic group.

			**Akonolinga**				**Bankim**		
			**River**	**Flooded Area**	**Swamp**	**Stream**	**Flooded Area**	**Swamp**	**Stream**
**Vertebrates**	Fish		428 (0.9)	2 (0)	298 (0.3)	373 (0.9)	119 (0.9)	108 (0.8)	242 (2.6)
	Anura		2319 (4.6)	23 (0.1)	2533 (2.7)	941 (2.3)	76 (0.6)	1151 (8.4)	196 (2.1)
**Invertebrates**	Insecta	Odonata	1878 (3.8)	868 (4.7)	15663 (16.5)	5106 (12.7)	1636 (12.5)	2430 (17.8)	1756 (18.8)
		Ephemeroptera	8640 (17.3)	9979 (53.8)	17057 (17.9)	8198 (20.4)	3648 (27.9)	581 (4.2)	1180 (12.6)
		Hemiptera	6746 (13.5)	1635 (8.8)	6497 (6.8)	2441 (6.1)	1257 (9.6)	928 (6.8)	944 (10.1)
		Coleoptera	22180 (44.4)	2633 (14.2)	29549 (31.1)	1989 (4.9)	2073 (15.8)	2493 (18.2)	777 (8.3)
		Diptera	5287 (10.6)	2654 (14.3)	17392 (18.3)	5646 (14)	3684 (28.2)	3918 (28.6)	2391 (25.6)
		Trichoptera	168 (0.3)	10 (0.1)	532 (0.6)	2248 (5.6)	32 (0.2)	15 (0.1)	243 (2.6)
		Plecoptera	3 (0)	3 (0)	4 (0)	18 (0)	0 (0)	2 (0)	2 (0)
		Lepidoptera	64 (0.1)	60 (0.3)	116 (0.1)	132 (0.3)	10 (0.1)	33 (0.2)	83 (0.9)
	Mollusca	Gastropoda	214 (0.4)	46 (0.2)	1440 (1.5)	3115 (7.7)	95 (0.7)	1203 (8.8)	255 (2.7)
		Bivalvia	47 (0.1)	0 (0)	92 (0.1)	133 (0.3)	0 (0)	1 (0)	0 (0)
	Crustacea	Decapoda	199 (0.4)	5 (0)	12 (0)	7547 (18.8)	0 (0)	0 (0)	195 (2.1)
		Cladocera	451 (0.9)	11 (0.1)	458 (0.5)	27 (0.1)	8 (0.1)	258 (1.9)	13 (0.1)
	Annelida	Hirudinea	214 (0.4)	114 (0.6)	613 (0.6)	893 (2.2)	52 (0.4)	142 (1)	487 (5.2)
		Oligochaeta	168 (0.3)	262 (1.4)	266 (0.3)	353 (0.9)	19 (0.1)	75 (0.5)	31 (0.3)
	Arachnida	Acari	523 (1)	75 (0.4)	1073 (1.1)	109 (0.3)	155 (1.2)	28 (0.2)	91 (1)
		Araneae	407 (0.8)	134 (0.7)	1368 (1.4)	746 (1.9)	189 (1.4)	291 (2.1)	388 (4.2)
	Collembola		66 (0.1)	19 (0.1)	195 (0.2)	234 (0.6)	31 (0.2)	20 (0.1)	56 (0.6)
