# Supplementary material for: Mycobacterium ulcerans Ecological Dynamics and Its Association with Freshwater Ecosystems and Aquatic Communities: Results from a 12-Month Environmental Survey in Cameroon
Source: PLoS Negl Trop Dis. 2014 May 15;8(5):e2879. doi: 10.1371/journal.pntd.0002879 (PMC4022459; doi:10.1371/journal.pntd.0002879)
Supplement: Table S3 — Detection of M. ulcerans DNA by qPCR with KR and IS2404 sequences. Results are given for pools of aquatic vertebrates and invertebrates from Akonolinga (12 months of sampling) and Bankim (4 months of sampling). All pools were tested for the KR sequence. KR positive pools where then confirmed by IS2404. Only sample-pools positive to both sequences were considered positive. (PDF) [file pntd.0002879.s009.pdf]

Table S3. Detection of *M. ulcerans* DNA by qPCR with KR and IS2404 sequences. Results are given for pools of aquatic vertebrates and invertebrates from Akonolinga (12 months of sampling) and Bankim (4 months of sampling). All pools were tested for the KR sequence. KR positive pools where then confirmed by IS2404. Only pools positive to both sequences were considered positive.

			**Akonolinga**			**Bankim**		
			**KR+ / Total**	**IS24+ / KR+**	**KR+ & IS24+ /**	**KR+ / Total**	**IS24+ / KR+**	**KR+ & IS24+ /**
**Vertebrates**	Fish		9/66 (13.6)	6/9 (66.7)	6/66 (9.1)	6/57 (10.5)	5/6 (83.3)	5/57 (8.8)
	Anura		8/102 (7.8)	6/8 (75)	6/102 (5.9)	7/47 (14.9)	4/7 (57.1)	4/47 (8.5)
**Invertebrates**	Insecta	Odonata	29/242 (12)	18/29 (62.1)	18/242 (7.4)	16/120 (13.3)	16/16 (100)	16/120 (13.3)
		Ephemeroptera	26/239 (10.9)	12/26 (46.2)	12/239 (5)	15/118 (12.7)	14/15 (93.3)	14/118 (11.9)
		Hemiptera	39/263 (14.8)	32/39 (82.1)	32/263 (12.2)	13/133 (9.8)	13/13 (100)	13/133 (9.8)
		Coleoptera (Adults)	20/238 (8.4)	8/20 (40)	8/238 (3.4)	13/115 (11.3)	11/13 (84.6)	11/115 (9.6)
		Coleoptera (Larvae)	5/84 (6)	5/5 (100)	5/84 (6)	8/76 (10.5)	6/8 (75)	6/76 (7.9)
		Diptera	30/269 (11.2)	24/30 (80)	24/269 (8.9)	18/144 (12.5)	15/18 (83.3)	15/144 (10.4)
		Trichoptera	1/70 (1.4)	0/1 (0)	0/70 (0)	0/19 (0)	0/0 (0)	0/19 (0)
		Plecoptera	0/0	0/0	0/0	1/2 (50)	1/1 (100)	1/2 (50)
		Lepidoptera	1/20 (5)	1/1 (100)	1/20 (5)	6/24 (25)	5/6 (83.3)	5/24 (20.8)
	Mollusca		8/67 (11.9)	4/8 (50)	4/67 (6)	3/83 (3.6)	1/3 (33.3)	1/83 (1.2)
	Crustacea	Decapoda	3/45 (6.7)	3/3 (100)	3/45 (6.7)	0/2 (0)	0/0 (0)	0/2 (0)
		Cladocera	0/10 (0)	0/0 (0)	0/10 (0)	2/11 (18.2)	2/2 (100)	2/11 (18.2)
	Annelida		4/60 (6.7)	4/4 (100)	4/60 (6.7)	11/62 (17.7)	11/11 (100)	11/62 (17.7)
	Arachnida	Acari	4/59 (6.8)	3/4 (75)	3/59 (5.1)	0/48 (0)	0/0 (0)	0/48 (0)
		Araneae	9/81 (11.1)	5/9 (55.6)	5/81 (6.2)	5/79 (6.3)	4/5 (80)	4/79 (5.1)
	Collembola		0/16 (0)	0/0 (0)	0/16 (0)	0/12 (0)	0/0 (0)	0/12 (0)
