# Supplementary material for: Mycobacterium ulcerans Ecological Dynamics and Its Association with Freshwater Ecosystems and Aquatic Communities: Results from a 12-Month Environmental Survey in Cameroon
Source: PLoS Negl Trop Dis. 2014 May 15;8(5):e2879. doi: 10.1371/journal.pntd.0002879 (PMC4022459; doi:10.1371/journal.pntd.0002879)
Supplement: Table S4 — Distribution of M. ulcerans positive macro-invertebrates and vertebrates over space and time. Values indicate the number of sites and months where a taxonomic group has been found positive to M. ulcerans DNA by both IS2404 and KR out of the total number of sites and months where the group has been tested. (PDF) [file pntd.0002879.s010.pdf]

Table S4: Distribution of *M. ulcerans* positive macro-invertebrates and vertebrates over space and time. Values indicate the number of sites and months where a taxonomic group has been found positive to *M. ulcerans* DNA by both IS2404 and KR out of the total number of sites and months where the group has been tested.

			Sites positive/Sites tested (%)	Months positive/Months tested (%)
**Vertebrates**	Fish		6/27 (22)	6/12 (50)
	Anura		6/20 (30)	5/12 (42)
**Invertebrates**	Insecta	Odonata	16/32 (50)	8/12 (67)
		Ephemeroptera	12/32 (38)	7/12 (58)
		Hemiptera	21/32 (66)	11/12 (92)
		Coleoptera (Adults)	10/32 (31)	6/12 (50)
		Coleoptera (Larvae)	5/16 (31)	4/5 (80)
		Diptera	19/32 (59)	9/12 (75)
		Trichoptera	0/17 (0)	0/12 (0)
		Plecoptera	1/1 (100)	1/1 (100)
		Lepidoptera	3/13 (23)	2/5 (40)
	Mollusca		3/24 (13)	3/12 (25)
	Crustacea	Decapoda	1/8 (13)	2/11 (18)
		Cladocera	1/7 (14)	1/2 (50)
	Annelida		8/19 (42)	5/11 (45)
	Arachnida	Acari	1/14 (7)	2/8 (25)
		Araneae	5/21 (24)	4/9 (44)
	Collembola		0/11 (0)	0/4 (0)
